# Potential Use of Biosensors for the Rapid and Specific Isolation of *Listeria monocytogenes* from Ready-to-Eat (RTE) Foods

**DOI:** 10.3390/pathogens14121280

**Published:** 2025-12-12

**Authors:** McCoy Williams, Rawah Faraj, Rejoice Nyarku, Savannah Simon, Kingsley E. Bentum, Ahmed Ghazy, Yilkal Woube, Temesgen Samuel, Evangelyn Alocija, Woubit Abebe

**Affiliations:** 1 Center for Food Animal Health, Food Safety, and Food Defense, College of Veterinary Medicine, Tuskegee University, Tuskegee, AL 36088, USA; mwilliams1720@tuskegee.edu (M.W.); rfaraj@tuskegee.edu (R.F.); rnyarku8794@tuskegee.edu (R.N.); ssimon1743@tuskegee.edu (S.S.); kbentum8786@tuskegee.edu (K.E.B.); ywoube@tuskegee.edu (Y.W.); tsamuel@tuskegee.edu (T.S.); 2 Veterinary Services Department of Egyptian Armed Forces, Cairo 11768, Egypt; ahmed.ghazy366@gmail.com; 3 Food Hygiene and Control Department, Faculty of Veterinary Medicine, Sadat University, Sadat City 32897, Egypt; 4 Global Alliance for Rapid Diagnostics, Michigan State University, East Lansing, MI 48824, USA; alocilja@msu.edu

**Keywords:** colorimetric, biosensor, magnetic nanoparticles, gold nanoparticles, *Listeria monocytogenes*

## Abstract

*Listeria monocytogenes* is a major foodborne pathogen associated with increasing global public health concern due to numerous outbreaks. Rapid pathogen detection is critical for reducing both the incidence and severity of foodborne illnesses. Recent advances in nanotechnology are transforming analytical methods, particularly for detecting foodborne pathogens. Magnetic nanoparticles (MNPs) and gold nanoparticles (GNPs) are among the most widely used nanomaterials in this field. This study investigated the potential use of MNPs and GNPs for the rapid and specific isolation of *L. monocytogenes* from fresh salad, deli meat, and frozen vegetables. *L. monocytogenes* (ATCC 19117) served as the model organism for biosensing and target capture. Results showed that the limits of detection (LoDs) for the GNP-based plasmonic/colorimetric biosensor and the MNP-based biosensor were 2.5 ng/µL DNA and 1.5 CFU/mL, respectively. Both GNPs and MNPs specifically detected *L. monocytogenes* even in the presence of closely related pathogens. Integration of MNPs and GNPs significantly enhanced the sensitivity of *L. monocytogenes* detection. Within one hour, naturally contaminated pre-packaged salad samples demonstrated clear evidence of effective direct capture by MNPs and specific identification by GNPs. This combined approach enables rapid and accurate on-site detection of *L. monocytogenes*, facilitating timely intervention and reducing the risk of contaminated foods reaching consumers.

## 1. Introduction

*Listeria monocytogenes* is a major foodborne pathogen responsible for invasive listeriosis and gastroenteritis, with symptoms ranging from mild gastrointestinal illness to septicemia, meningitis, abortion, and death [1]. Although infections are relatively rare, the case fatality rate approaches 20–30% and disproportionately affects the elderly and immunocompromised [1]. In the United States, an estimated 1600 infections occur annually, nearly 20% of which are fatal [1], resulting in substantial economic losses estimated at USD 1.282 million per case and USD 2.04 × 10^12^ overall [2]. Outbreaks also lead to major financial disruptions, including 22–27% declines in sales of ready-to-eat (RTE) foods following recalls [3,4]. Preventing contaminated products from entering the market therefore remains a public health and economic priority.

*L. monocytogenes* is ubiquitous in nature and can enter the food chain through environmental reservoirs or contaminated farm animals [5]. Its ability to grow at refrigeration temperatures, tolerate wide pH and water activity ranges, and form biofilms in food processing facilities contributes to its persistence and contamination of food-contact surfaces [6]. RTE foods are at particular risk because they receive no further antimicrobial treatment prior to consumption [7]. Although the infective dose is generally considered high (>10^4^ CFU/g) [8], susceptible individuals may develop illness at levels as low as 10^2^–10^4^ CFU/g [9,10]. Prevalence studies have shown contamination in 10% of frozen vegetable samples in England [11], and a major U.S. outbreak in 2016 linked to IQF vegetables resulted in nine illnesses, three deaths, and extensive product recalls [12,13,14]. These events highlight the need for rapid, reliable detection methods that can prevent contaminated foods from reaching consumers [15].

Accurate detection remains central to surveillance and outbreak response. Traditional culture-based methods provide high sensitivity (1–5 CFU/test portion) [16,17] but are slow and labor-intensive and require specialized laboratory infrastructure. Rapid assays, including lateral flow tests, ELISA, and PCR, offer improved speed and specificity [16] but remain limited by cost, potential false positives [6], matrix interference, and dependence on trained operators and well-equipped laboratories [6,18,19]. Many workflows are further hindered by food matrix complexity [20], low pathogen loads that require 8–18 h of enrichment before detection [21], and narrow testing budgets within the food industry [22]. Although magnetic bead-based concentration methods can improve bacterial recovery, they often yield variable results and may be affected by food debris [16,23,24,25,26]. Immunomagnetic nanoparticles improve specificity but are constrained by antibody cost, limited stability, and reduced performance in complex matrices [27,28].

Carbohydrate-coated magnetic nanoparticles (gMNPs) offer a cost-effective, robust, and broadly reactive alternative to antibody-based capture methods [29]. These nanoparticles demonstrate long shelf life, stability, and compatibility across diverse food matrices and can be paired with downstream biosensing platforms to achieve high sensitivity without the need for expensive or fragile recognition ligands [30,31,32,33,34,35]. Biosensor technologies, particularly optical systems, for the detection of *L. monocytogenes* provide rapid, real-time, and on-site analytical capabilities for food safety monitoring [36]. In these systems, interactions between bioreceptors (nucleic acids, antibodies, enzymes, or whole cells) and target molecules are converted into measurable physical signals through optical, electrochemical, or mass-based transducers [37]. Gold nanoparticle (GNP)-based optical biosensors are especially promising due to their strong surface plasmon resonance (SPR), which enables highly sensitive detection measurable by UV–Vis spectrophotometry [38,39].

**Figure 1:**
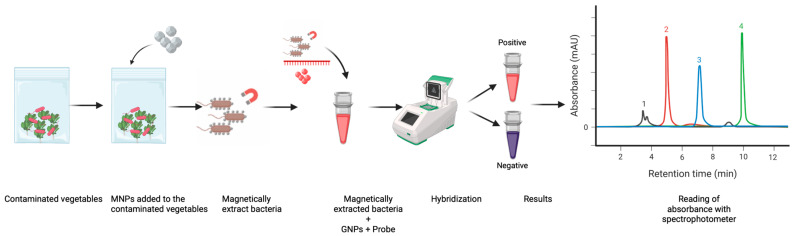
Workflow of magnetic nanoparticle–assisted bacterial capture and colorimetric detection from produce. Produce samples are mixed with functionalized MNPs, allowing selective binding to bacterial cells, followed by magnetic separation. Bound bacteria react with GNP-based probes to generate positive (red) or negative (purple) colorimetric outputs, which are quantified spectrophotometrically and visualized as chromatographic peaks.

Despite these advances, several limitations persist. Traditional pathogen extraction workflows rely on recognition ligands requiring cold storage, which increases cost and reduces practicality in low-resource settings; in contrast, glycan-coated MNPs circumvent this need and allow for rapid capture of large sample volumes [40]. Likewise, many GNP-based assays depend on DNA amplification or lengthy probe-functionalization steps that can exceed 24 h [41]. To address these constraints, the present study demonstrates a rapid, amplification-free method for detecting *L. monocytogenes* using a combination of MNP-based bacterial concentration and GNP-based colorimetric DNA detection in fresh salad, deli meat, and frozen vegetable matrices (Figure 1).

In this study, we aimed to address the need for rapid, sensitive, and field-deployable detection of *L. monocytogenes* in RTE foods. We identified a critical gap in the availability of low-cost biosensing systems capable of both concentrating and detecting pathogens without DNA amplification or antibody-based capture. We hypothesized that carbohydrate-coated magnetic nanoparticles (MNPs), used in combination with dextrin-capped gold nanoparticles (GNPs), could achieve rapid, specific, and highly sensitive detection of *L. monocytogenes* directly from complex food matrices. To test this hypothesis, our objectives were to: (i) evaluate the capture efficiency of MNPs in pure culture and food matrices; (ii) determine the sensitivity and specificity of the GNP-based biosensor; and (iii) validate the integrated MNP–GNP system using naturally contaminated food samples.

## 2. Materials and Methods

### 2.1. Reagents and Chemicals

Magnetic nanoparticles (MNPs) coated with chitosan and gold nanoparticles (GNPs) coated with dextrin were developed by the Alocilja Research Group at Michigan State University. The MNPs are shelf-stable at room temperature for at least 3 years, while the GNPs are stored at 4 °C and remain stable for at least 3 years. The GNP-MUDA formulation is also stored at 4 °C and is stable for at least 6 months. The MNPs, composed of iron oxide (magnetite), are coated with chitosan to facilitate efficient capture of bacterial cells. The chitosan-coated MNPs used in this study had an approximate core size of 50–80 nm, with an average stock concentration of 5 mg/mL. The dextrin-capped GNPs were approximately 20–25 nm in diameter and supplied at a concentration optimized for colorimetric assays. Nanoparticles were stored at 4 °C in the dark to prevent aggregation and equilibrated to room temperature prior to use. This cost-effective approach eliminates the need for expensive antibodies or aptamers commonly required in immunomagnetic separation, allowing the nanoparticles to interact effectively with glycoproteins on the bacterial cell surface [20].

### 2.2. Primer Design and Biosensor Probe

Primers previously reported in the literature were adopted, and the corresponding gene sequences were retrieved and processed using SnapGene^®^ software (GSL Biotech LLC, a Dotmatics company; Chicago, IL, USA, snapgene.com) for specific primer and probe design. The biosensor probe used in this study was Lmo0733-2F-Biosensor with the following sequence: /5AmMC6/TG GAA AGT TGT TTG CTC TTT CTT TTG TTG TTC TGC TGT ACG A.

### 2.3. Bacterial Culture

*Listeria monocytogenes* (ATCC 19117) was obtained from the Center for Food Animal Health, Food Safety and Defense Laboratory, Tuskegee University, USA. Cultures were grown in Listeria Enrichment Broth (LEB) (Hardy Diagnostics, Santa Maria, CA, USA) at 30 °C for 24 h and used throughout the study.

### 2.4. Spiking of PBS and Salad Samples to Determine the Limit of Detection (LoD) of MNPs and GNPs

The limits of detection (LoD) of the MNP and GNP biosensors were evaluated in phosphate-buffered saline (PBS) and salad samples through spiking and recovery experiments. *L. monocytogenes* (ATCC 19117) was cultured in LEB at 30 °C for 24 h and centrifuged to obtain a bacterial pellet. The supernatant was discarded, and the pellet was resuspended in 1× PBS to a 0.5 McFarland standard (1.5 × 10^8^ CFU/mL), corresponding to 0.1 absorbance on a turbidimeter.

Salad samples were first exposed to UV light for 30 min to eliminate naturally occurring *L. monocytogenes*. Ten grams of UV-treated salad were weighed into a Whirl-Pak bag and spiked with 1 mL of the 0.5 McFarland suspension. Nine milliliters of PBS were then added, and the mixture was homogenized in a stomacher for 2 min, yielding a concentration of 1.5 × 10^8^ CFU/mL.

**Figure 2:**
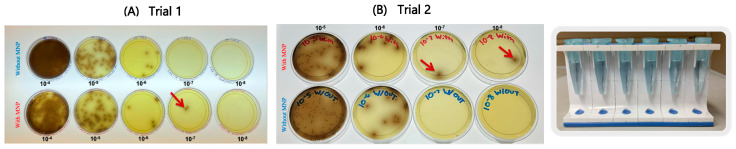
Serial tenfold dilutions (10^−4^–10^−8^) of ATCC *Listeria monocytogenes* in PBS plated on Oxford agar (**A**,**B**). Results represent two independent experimental trials. Red arrows indicate the capture efficiency of MNPs at the lowest detectable dilution level.

From this homogenate, 100 µL was transferred into 900 µL of PBS (1.5 × 10^7^ CFU/mL), followed by 10-fold serial dilutions from 10^−1^ (1.5 × 10^7^ CFU/mL) to 10^−8^ (1.5 CFU/mL). These serial dilutions were prepared in three sets (Figure 2):

Set A: without MNPs

Set B: with MNPs

Set C: for GNP LoD experiments

Dilutions from 10^−4^ to 10^−8^ were used in all experiments. Uninoculated salad samples served as negative controls. The limit of detection (LoD) was defined as the lowest dilution at which *L. monocytogenes* was consistently detected in at least 95% (≥19/20) of replicate reactions. All experiments, including spiking trials, capture efficiency assays, GNP sensitivity and specificity tests, and naturally contaminated sample analyses, were performed in triplicate unless otherwise stated. Mean values were used for all calculations.

### 2.5. Capture Efficiency of MNPs

Capture efficiency was assessed using the two sets of serial dilutions described in Section 2.4 (Set A: without MNPs; Set B: with MNPs). The experiment was based on the method described by [21] with modifications.

For Set B, 10 µL of 5 mg/mL MNPs were added to each serially diluted sample (10^−4^ to 10^−8^). Tubes were incubated in a shaker at 32 °C for 10 min and then placed on a magnetic rack for 5 min to pellet the MNP-bacteria complexes. The supernatant was carefully removed without disturbing the complexes, which were then resuspended in 90 µL of PBS. The entire resuspension was plated on Listeria Oxford agar and incubated at 37 °C for 24 h.

For Set A, 20 µL of each dilution was directly plated without MNP treatment to determine the initial CFU/mL. Colonies were enumerated for all dilutions, and capture efficiency (CE) was calculated as [21]:

$$CE\left(\%\right)=\frac{Log10\left(captured\;cells\;CFU/mL\right)}{\mathrm{Log}10\left(cell\;count\;before\;capture\;CFU/mL\right)}*100$$

Experiments were performed twice, and mean colony counts were used for CE calculations.

### 2.6. Analytical Specificity of GNP Biosensor for Detecting L. monocytogenes

*L. monocytogenes* (ATCC 19117) was grown in LEB at 30 °C for 24 h, pelleted by centrifugation, and subjected to DNA extraction using the DNeasy Blood & Tissue Kit (Qiagen, Germantown, MD, USA). Extracted DNA was used in the GNP biosensor assay.

To evaluate specificity, *Escherichia coli* O157:H7 DNA was extracted and included as a non-target control. The GNP protocol followed Dester et al. [40]. Briefly, 5 µL GNPs, 5 µL probe, and 10 µL DNA were combined in a 0.2 mL tube. Hybridization was performed in a thermocycler with the following conditions: 95 °C for 5 min (denaturation); 55 °C for 10 min (annealing); 4 °C for 5 min (cooling).

Then, 3 µL of 0.1 M HCl were added gradually, and colorimetric results were read after 5–10 min. Target DNA remained red, whereas non-target DNA aggregated and turned purple/blue. Absorbance at 520 nm was measured using a NanoDrop™ 2000C spectrophotometer (Thermo Fisher Scientific; Wilmington, DE, USA).

### 2.7. Assessment of MNP and GNP Biosensor Combination in Naturally Contaminated Food Samples

A total of 50 food samples, including premade salad, frozen spinach, frozen mixed vegetables, frozen Brussels sprouts, deli meat, queso fresco, and RTE fresh lettuce were collected from Tuskegee and Auburn, Alabama, USA.

Presence of *L. monocytogenes* was confirmed using the FDA BAM method. Briefly, 10 g of each sample were mixed with 100 mL Buffered Peptone Water and shaken for 30 s at 250 rpm, then incubated for 24 h at 37 °C. One hundred microliters of the suspension were transferred to LEB and incubated for 48 h at 30 °C. Cultures were plated onto BIO-RAD Agar and Oxford Agar, then incubated for 48 h at 37 °C. A rhamnose biochemical test was performed for confirmation.

For biosensor analysis, 10 g of each sample were mixed with 100 mL Buffered Peptone Water in a Whirl-Pak bag and homogenized for 30 s at 250 rpm. Then, 10 µL of 5 mg/mL MNPs were added and incubated in a shaker for 10 min. Samples were placed on a magnetic rack for 5 min, and the captured MNP-bacteria complex was resuspended in 90 µL PBS.

For detection, 5 µL GNPs, 5 µL probe, and the MNP-bacteria complex were combined and subjected to the same thermocycling and HCl-induced colorimetric protocol described in Section 2.6. PCR was performed on extracted genomic DNA to confirm that GNP stability corresponded to the presence of *L. monocytogenes* DNA.

### 2.8. Data Analysis

A two-way ANOVA was used to compare means of *L. monocytogenes* cells captured bsy MNPs in spiked PBS and spinach samples using GraphPad Prism version 10.3.0. Significance was set at α = 0.05.

## 3. Results

### 3.1. Spiking of PBS and Salad Samples to Determine the LoD of MNPs and GNPs

**Figure 3:**
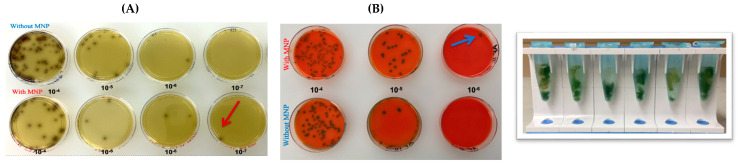
Serial tenfold dilutions (10^−4^–10^−8^) of ATCC *L. monocytogenes* inoculated onto spinach and subsequently plated on Oxford agar (**A**) and RAPID’ *L. mono* agar (Bio-Rad, Hercules, CA, USA) (**B**). Red and blue arrows indicate the capture efficiency of MNPs at the lowest detectable dilution level.

Serial dilution experiments performed in PBS (Figure 2) demonstrated that the MNPs successfully captured *L. monocytogenes* down to the 10^−7^ and 10^−8^ dilutions, equivalent to concentrations of approximately 15 CFU/mL and 1.5 CFU/mL, respectively. Similarly, experiments conducted with spinach samples (Figure 3) showed that the MNPs captured *L. monocytogenes* as low as the 10^−7^ dilution (15 CFU/mL per 10 g of food). These findings confirm that the MNPs maintained strong capture capability even in a complex food matrix.

### 3.2. MNPs Capture Efficiency Results

Capture efficiency results demonstrated robust performance of MNPs in both PBS and spinach samples. In PBS, the capture efficiency ranged from 69% to 71%. At the lowest dilutions tested (10^7^ and 10^8^, corresponding to 15 CFU/mL and 1 CFU/mL), one colony was captured using MNPs, whereas no colonies were observed in corresponding dilutions without MNPs.

**Figure 4:**
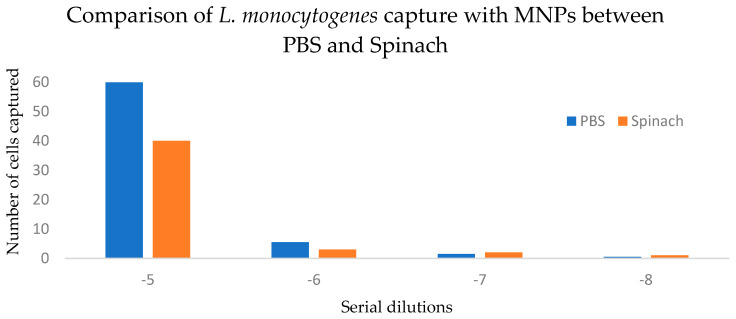
Comparison of *L. monocytogenes* capture with MNPs between spiked PBS and spinach samples. *p* > 0.05 (0.355).

In spinach, capture efficiency ranged from 66% to 90% (Figure 4), highlighting the ability of MNPs to function effectively even in food matrices containing natural debris and inhibitory substances.

Two-way ANOVA without replication showed a significant effect of dilution level on capture efficiency (*p* = 0.0138), accounting for 94.4% of total variation. The matrix (PBS vs. spinach) did not significantly affect capture efficiency (*p* = 0.355), contributing only 1.6% of the variation. Because measurements were not replicated within each matrix-by-dilution combination, interaction effects could not be tested. The 95% confidence interval for the mean difference in capture efficiency between PBS and spinach was −10.30 to 21.05, indicating no statistically significant difference between matrices (Table 1).

### 3.3. Analytical Sensitivity of GNP Biosensor for Detecting L. monocytogenes

**Figure 5:**
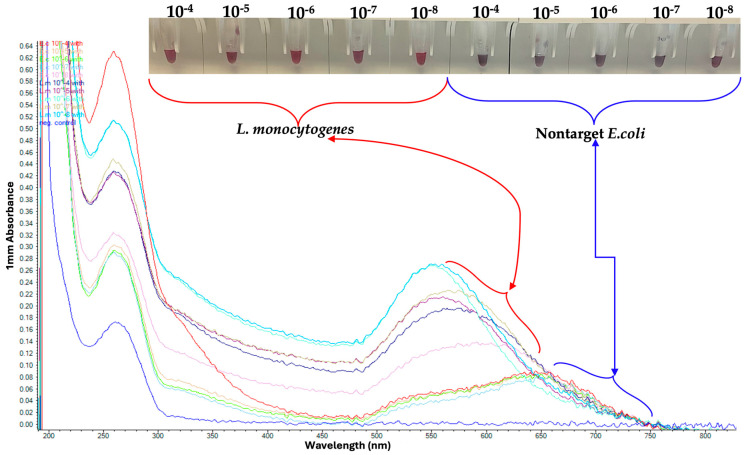
Serial Dilutions of *L. monocytogenes* tested on Gold Nanoparticle (GNP). Red coloration indicates dispersed GNPs in the presence of *L. monocytogenes* DNA, whereas purple/blue coloration represents nanoparticle aggregation in the absence of target DNA.

**Figure 6:**
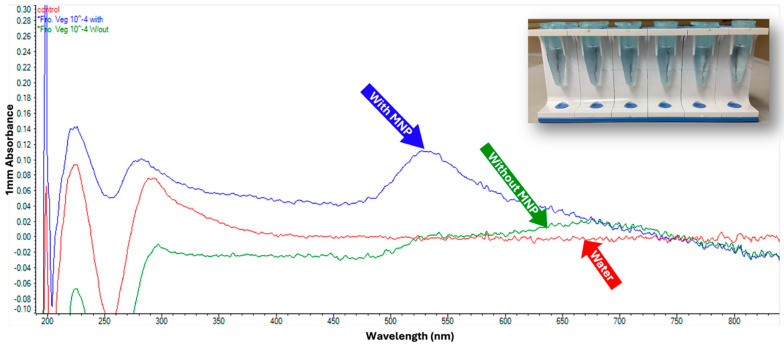
GNP testing on *L. monocytogenes* without DNA extraction. This assay demonstrates that GNPs can detect released bacterial DNA following thermal lysis without the need for DNA extraction.

Using a range of DNA concentrations, the GNP biosensor effectively detected purified *L. monocytogenes* DNA at concentrations as low as 2.5 ng/µL, even in the presence of non-target *E. coli* DNA, as shown in Figure 5 and Figure 6. These results demonstrate both the high sensitivity and the robustness of the GNP-based detection platform.

### 3.4. Observed Analytical Specificity of GNP Biosensor for Detecting L. monocytogenes

Following magnetic capture using MNPs, the GNP biosensor successfully detected *L. monocytogenes* across dilutions from 10^−4^ to 10^−8^, corresponding to concentrations as low as 1 CFU/mL (Figure 5). The assay clearly differentiated *L. monocytogenes* from the non-target pathogen *E. coli*, confirming high analytical specificity.

### 3.5. Assessment of MNP and GNP Biosensors in Naturally Contaminated Food Samples

Among the 50 food samples tested, 13 were positive for *L. monocytogenes* and were subjected to further analysis. The premade salad category (samples PW, AD, AS) showed the highest positivity rate of 60% (3/5). In the frozen food category, frozen mixed vegetables exhibited a positivity rate of 33.3% (1/3), while both frozen spinach and frozen Brussels sprouts tested negative.

Single samples of deli meat, queso fresco, and RTE fresh lettuce all tested negative (0%).

**Figure 7:**
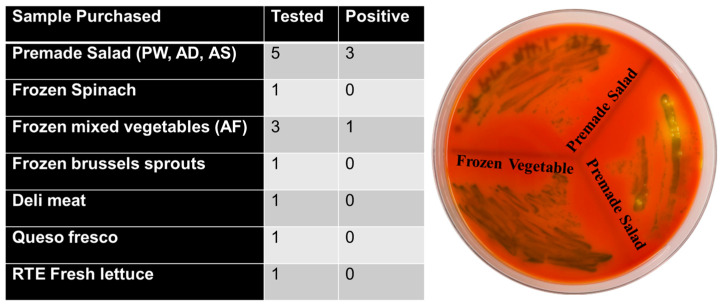
*Listeria monocytogenes* detection in RTE food samples. Table summarizes the number of food items tested and those positive for *L. monocytogenes*. Premade salads showed 3 positives out of 5 samples, and one frozen mixed vegetable sample also tested positive. All other products were negative. (Right) Representative chromogenic agar plate displaying typical dark green/black *L. monocytogenes* colonies recovered from premade salad and frozen mixed vegetable samples.

**Figure 8:**
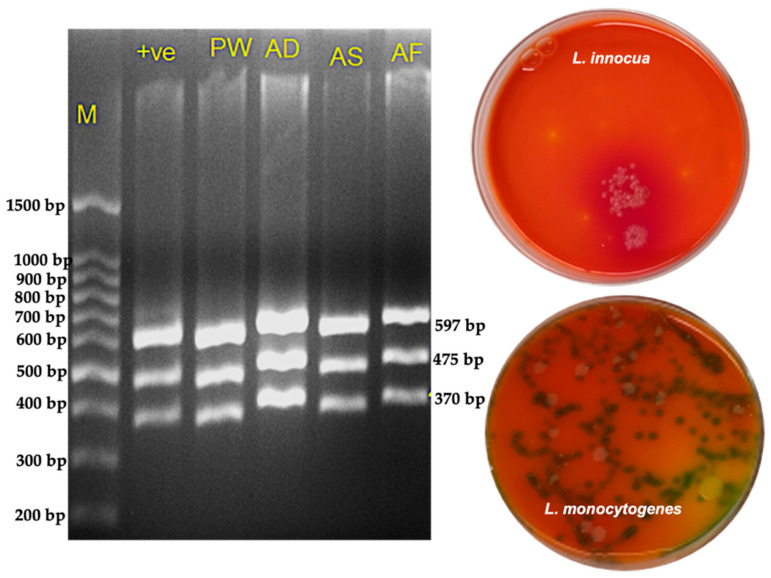
Confirmation of *Listeria* isolates by PCR and selective plating. (**Left**) Agarose gel electrophoresis showing amplification profiles of *Listeria monocytogenes* premade salad (PW, AD, AS) and frozen mixed vegetables (AF). Lane M: molecular weight marker; Lane +ve: positive control (*L. monocytogenes* serogroup 4 d, ATCC strain). All test lanes display the characteristic multi-band pattern consistent with the expected *L. monocytogenes* target fragments. (**Right**) Selective plating on chromogenic medium illustrating phenotypic differentiation between *L. innocua* and *L. monocytogenes*. *L. innocua* colonies (top plate) appear smooth, pale, and non-pigmented, lacking the characteristic color change. *L. monocytogenes* colonies (bottom plate) exhibit the typical darker, bluish-green to black colonies indicative of β-glucosidase activity. The combined molecular and culture-based results confirm accurate identification and discrimination of *Listeria* species from RTE food samples.

Across all sample types, the overall positivity rate was 30.8%, with premade salads showing the greatest incidence of contamination (show in Figure 7). PCR verification of positive samples is shown in Figure 8.

## 4. Discussion

The results of this study demonstrate the effectiveness of magnetic nanoparticles (MNPs) and gold nanoparticles (GNPs) for capturing and detecting *Listeria monocytogenes* in a variety of food matrices, including phosphate-buffered saline (PBS) and spinach. The findings highlight the potential of these nanoparticle-based biosensing methods as rapid, sensitive, and cost-effective alternatives to conventional pathogen detection techniques. Several biosensor platforms have previously been used for the detection of *L. monocytogenes* (Table 2), and the present study contributes to this growing body of evidence.

The ability of MNPs to capture *L. monocytogenes* at extremely low concentrations down to dilutions as high as 10^−7^ and 10^−8^ in PBS, demonstrates the high sensitivity of the method. These dilutions correspond to bacterial concentrations of approximately 15 CFU/mL and 1.5 CFU/mL, respectively. Given that the infectious dose for healthy individuals is estimated at 10 to 100 million CFU, and as low as 0.1 to 10 million CFU for individuals at higher risk [42], the sensitivity of this biosensing system is sufficient to support early detection and prevention of listeriosis. Early identification is essential for minimizing the spread of contamination and preventing foodborne outbreaks. The significant effect of dilution level observed in the ANOVA is biologically expected, as nanoparticle bacteria collision probability decreases with declining microbial load. This supports the sensitivity profile of the MNP system and aligns with kinetic interaction models demonstrating reduced capture rates at lower bacterial concentrations.

Capture efficiency results further support the robustness of the MNP platform. In PBS, capture efficiency ranged from 69% to 71%, while in spinach, a more complex matrix containing natural inhibitors, it ranged from 66% to 90%. This performance indicates that MNPs can function effectively across sample types and withstand the challenges posed by food matrices. Importantly, the ability to detect *L. monocytogenes* in spinach at concentrations as low as 15 CFU/mL illustrates the real-world applicability of this approach to ready-to-eat (RTE) foods, where low levels of contamination can pose significant risks due to the absence of a kill step prior to consumption. Statistical analysis further demonstrated that the matrix (PBS vs. spinach) did not significantly influence capture efficiency (*p* = 0.355). The 95% confidence interval for the mean difference between matrices (−10.30 to 21.05) included zero, indicating that any true difference is likely small. This suggests that moderate matrix-associated inhibitors present in leafy greens do not substantially interfere with glycan–nanoparticle interactions. These findings align with previous reports showing that carbohydrate-functionalized magnetic nanoparticles retain performance in the presence of fibers, pigments, and polyphenols commonly found in plant-based foods.

The strong capture performance observed in this study is consistent with known mechanisms of bacterial interaction with carbohydrate-coated magnetic nanoparticles. Prior work has shown that glycan-functionalized MNPs bind to Gram-positive bacteria through a combination of electrostatic attraction, hydrophobic interactions, and glycan-mediated adhesion. The negatively charged teichoic acids and peptidoglycan components of the *Listeria* cell wall interact with hydrophilic or positively charged carbohydrate ligands on the nanoparticle surface, while lectin-like domains on bacterial surfaces can engage mannose-, galactose-, and glucan-based ligands [30,31,32,33,34,35]. In addition, hydrophobic regions of the bacterial envelope promote adsorption via van der Waals and hydrophobic forces, facilitating rapid formation of bacteria nanoparticle complexes even in complex food matrices. These mechanisms help explain the high capture efficiencies observed across different sample types in this study.

The GNP-based biosensor also demonstrated strong analytical performance, exhibiting high specificity and sensitivity. The assay successfully distinguished *L. monocytogenes* from the non-target organism *Escherichia coli*, minimizing the likelihood of false-positive results, an important advantage in food safety testing where unnecessary recalls can have substantial economic impacts [43]. The GNP biosensor detected *L. monocytogenes* at levels as low as 1 CFU/mL following magnetic concentration, underscoring its capability for sensitive downstream detection. Notably, the limit of detection (LoD) reported in this study contrasts with previous findings by Xiao et al. [44], who reported an LoD of 10^2^ CFU/mL in lettuce samples, highlighting the improved sensitivity achieved using the combined MNP–GNP system.

The performance of the nanoparticle system in naturally contaminated food samples further demonstrates its practical applicability. Of the 50 samples tested, 13 were positive for *L. monocytogenes*, with premade salads exhibiting the highest positivity rate (60%). This aligns with previous reports indicating that RTE salads are among the highest-risk products for *Listeria* contamination [45,46]. The detection of *L. monocytogenes* in frozen mixed vegetables is consistent with the documented persistence of this pathogen in frozen foods, such as those implicated in the 2016 multistate outbreak involving individually quick-frozen (IQF) vegetables [13]. Negative results in deli meat, queso fresco, and RTE lettuce samples could be due to the absence of contamination or the limited number of samples tested. Food matrices naturally contain inhibitors such as fats, fibers, and polyphenols that can interfere with nanoparticle binding and optical responses. The ability of the MNP–GNP system to detect *L. monocytogenes* in spinach and premade salads demonstrates tolerance to moderate matrix interference; however, future studies should quantify the influence of pH, fat content, and particulate debris on capture efficiency. The lack of a significant matrix effect is advantageous for practical applications, as it indicates that the MNP system can deliver consistent performance across both simple (PBS) and complex (spinach) matrices. This level of robustness distinguishes the glycan-coated MNPs from antibody-based magnetic beads, which frequently exhibit reduced efficiency in complex samples due to steric hindrance or nonspecific competitive binding. However, the present findings do not rule out the possibility that highly fatty, viscous, or protein-rich matrices may impose stronger inhibitory effects. Additional validation across a broader range of RTE food products will clarify the operational limits of the technology.

Compared to traditional culture-based methods, the nanoparticle-based approach presented here offers several significant advantages. Although culture methods remain the gold standard for Listeria detection, they are time-consuming, labor-intensive, and require trained personnel and specialized facilities [47]. Moreover, conventional methods typically detect *L. monocytogenes* at levels ranging from 5 to 100 CFU per 25 g of food [48], while the combined MNP–GNP system used in this study achieved substantially lower detection limits. Importantly, the nanoparticle-based workflow can substantially reduce or eliminate the need for lengthy pre-enrichment steps, enabling faster decision-making during routine testing and outbreak response.

Detection with GNPs can be completed in approximately 30 min, and pathogen capture with MNPs requires about 15 min. With downstream confirmation through PCR or culture, the entire workflow can be completed within 1–24 h, markedly faster than conventional protocols. In addition, nanoparticle technology allows a single operator to perform the full detection process efficiently, reducing labor demands and overall operational costs. The assay is also highly cost-effective, with an estimated cost of less than USD 0.01 per reaction. Minimal equipment requirements further support its use as a point-of-care diagnostic tool [49].

A previous study reported the use of biofunctionalized magnetic nanoparticles with nuclear magnetic resonance (NMR) for rapid detection of *L. monocytogenes* in food samples. Although the NMR-based method required only 40 min, compared to the five days required by some national standards, the test cost remained relatively high at approximately USD 38 per assay, with detection limits ranging from 3 to 10^3^ CFU/mL [16]. In contrast, the system used in the present study offers enhanced sensitivity at significantly lower cost, making it more suitable for widespread adoption in food safety monitoring.

Overall, the results demonstrate that the combined use of MNPs and GNPs represents a highly promising approach for rapid and sensitive detection of *L. monocytogenes* in diverse food matrices. The method is well-suited for routine monitoring, outbreak investigations, and on-site testing at food processing facilities, potentially improving food safety and reducing the risks associated with *Listeria* contamination. This nanoparticle-based workflow could complement existing regulatory detection protocols, including FDA BAM and ISO 11290 [50], by providing rapid on-site screening to enable earlier intervention during contamination events. Taken together, these findings confirm that dilution level but not matrix drives variability in capture efficiency, reinforcing the robustness and practical suitability of the MNP platform for diverse food safety applications.

**Table 2:** Summary of biosensing Strategies for *Listeria monocytogenes*.

Recognition Molecule/Nanomaterial	Sample Matrix	Detection Time	LOD	Method Type	Ref.
Glycan-coated MNPs + dextrin-capped GNPs (This study)	RTE foods	3 h	1.5 CFU/mL (MNP); 2.5 ng/µL DNA (GNP)	Magnetic capture + plasmonic colorimetric	This study
CRISPR/Cas12a (RAA-CRISPR platform)	Pure culture, genomic DNA	20–30 min	350 CFU/mL; 5.4 × 10^−3^ ng/µL	CRISPR fluorescence	[51]
High-resolution melting (HRM) qPCR	Roast pork	3–6 h	6.2 × 10^3^–6.2 × 10^4^ CFU/mL	mPCR + HRM qPCR	[52]
PEG-cefepime MNPs	Lettuce, juices, meat	110 min	3.1 × 10^2^ CFU/mL	Antibiotic MNP + colorimetry	[44]
Antibody-ZIF-8 GOD@ZIF-8@Ab	Juice	NS	10^1^ CFU/mL	Colorimetry	[53]
Teicoplanin MNPs	PBS, ground beef	NS	2.6 × 10^1^ CFU/mL	Fluorescence	[54]
Ampicillin-MNPs + qPCR	Milk	2.5 h	10^2^ CFU/mL	qPCR	[55]
Aptamer-linked AuNP + MNP	Milk	NS	6 CFU/mL	Colorimetric immunoassay	[56]
Immunomagnetic beads + SERS	Milk	NS	12 CFU/mL	SERS	[57]
D-amino acid MNPs	Milk, meat	30 s	2.17 × 10^2^ CFU/mL	Colorimetric	[58]
Antibody–MNP + nitrocellulose	Vegetables	35 min	1 × 10^2^ CFU/g	Colorimetry	[59]
Fe3O4@silica antibody NPs	Pure culture	30 min	NS	Magnetic capture	[60]
AuNP + Ag nanoclusters + aptamer MNPs	Food	NS	10 CFU/mL	Colorimetric	[61]
Impedance immunosensor + MNPs	Lettuce, milk, beef	3 h	10^4^ CFU/mL	Impedance	[62]
Aptamer-MNP + AIE fluorescence	Spiked samples	NS	10 CFU/mL	Fluorescence	[63]
Vancomycin-PEG-MNPs + PCR	PBS, lettuce	<4 h	30 CFU/g	PCR	[64]
Fe/Fe3O4 NPs + antibodies (NMR)	Milk powder, lettuce	NS	3 MPN	NMR	[16]
CRISPR/Cas12a electrochemical	Plant samples	2 h	0.68 aM; 940 CFU/g	Electrochemical	[36]
Mesoporous silica microarray	Food samples	2 h	10^2^ CFU/mL	Microarray	[65]
Aptamer-MNP + AuNP amplification	Meat, milk	1.5 h	10 CFU/mL	Colorimetric	[66]
AuNP-SD-PMA-qPCR	Milk	6 h	5 × 10^1^ CFU/g	qPCR + viability dye	[67]
Aptamer-based MNP system	Food	18 h	10^3^ CFU/mL	Fluorescence	[68]
Boronate affinity magnetic + fluorescence	Lettuce	35 min	2.2 × 10^1^ CFU/mL	Fluorescence	[69]
Cefepime-PEG-MNPs + colorimetry	Lettuce, juice, meat	100 min	3.1 × 10^2^ CFU/mL	Colorimetric	[44]
G-quadruplex DNAzyme colorimetric	Pork	4 h	3.1 CFU/mL	Colorimetric	[70]
Lateral flow strip (end-on mAb)	Blood, milk, mushrooms	15 min	10^1^–10^4^ CFU/mL	Lateral flow	[71]
QCM aptasensor + MNPs	Milk, cheese, meats, vegetables	10 min	148 CFU/mL	QCM	[72]
LAMP electrochemical sensor	Multiple foods	30 min (post enrichment)	1 CFU/25g	LAMP + electrochemical	[73]
CPA isothermal amplification	Rice flour	60 min	10^4^ CFU/mL	CPA	[74]
AlphaLISA nucleic acid assay	Milk, juice	NS	250 attomole	AlphaLISA	[75]

Abbreviations: LOD, limit of detection; NS, not specified; CFU, colony-forming units; MNPs, magnetic nanoparticles; GNPs, gold nanoparticles; CRISPR, clustered regularly interspaced short palindromic repeats; HRM, high-resolution melting; SERS, surface-enhanced Raman scattering; PCR, polymerase chain reaction; QCM, quartz crystal microbalance; LAMP, loop-mediated isothermal amplification; CPA, crossing priming amplification.

### Limitations and Future Directions

While this study demonstrates the strong potential of MNPs and GNPs for the detection of *L. monocytogenes*, several limitations should be addressed in future research. One limitation is the non-specific nature of carbohydrate-coated MNPs. Although these particles provide a cost-effective alternative to antibody-based capture, their non-specificity may be a disadvantage in situations where multiple pathogens are present in the same sample, potentially complicating interpretation of results. Future work should therefore focus on improving the specificity of MNPs, for example, by incorporating multifunctional ligands capable of simultaneously capturing and differentiating among multiple pathogens. Selectivity in natural samples may also be enhanced by using pathogen-specific carbohydrate epitopes; for instance, biotinylated oligosaccharides have been successfully applied to streptavidin-coated magnetic beads to capture *E. coli* strains [32].

Another important direction is the integration of this nanoparticle-based detection approach with other rapid diagnostic technologies, such as microfluidics, automated processing systems, or portable biosensing devices. Such integration would enable high-throughput, hands-free screening of food samples with minimal operator input, reducing potential human error and enhancing testing efficiency. This would be especially beneficial in food production environments, where routine monitoring often requires processing large sample volumes within short time frames.

Overall, future development should aim to enhance the selectivity, automation, and scalability of the nanoparticle-based detection system. These improvements will support the broader adoption of rapid biosensing technologies in the food industry and increase their impact on food safety, public health, and outbreak prevention.

## 5. Conclusions

In conclusion, the combined use of magnetic nanoparticles (MNPs) and gold nanoparticles (GNPs) presents a highly promising alternative to traditional methods for detecting *Listeria monocytogenes* in food. This approach is highly sensitive, specific, and capable of identifying low levels of contamination across a variety of food matrices. Its rapid detection capability requiring minimal processing time and limited specialized equipment makes it particularly valuable for applications in the food industry, where timely identification of contamination is essential for preventing foodborne illness outbreaks. Continued research and development in this area may yield even more robust, selective, and versatile detection platforms, further enhancing food safety monitoring and contributing to improved public health protection.

## Figures and Tables

**Table 1:** Two-way ANOVA (Matrix × Dilution Level) for capture efficiency.

Source	SS	df	MS	% of Total Variation	*p*-Value	Interpretation
Dilution Level (10^−5^–10^−8^)	3433	3	1144.3	94.4%	0.0138	Significant
Matrix (PBS vs. Spinach)	57.8	1	57.8	1.6%	0.355	Not significant
Residual	145.6	3	48.5	4.0%	—	—

## Data Availability

Data are contained within this article.
